# Public Pharmacists' and Dispensers' Association

**Published:** 1913-11-08

**Authors:** 


### Public Pharmacists' and Dispensers' Association.

LECTURE ON FLIES AND DISEASE.

The inaugural meeting of the fifteenth session was held on October 29 at St. Bride Institute, Ludgate Circus, when Dr. T. S. Higgins, B.Sc., M.D., D.P.H., Medical Officer of Health, St. Pancras, lectured on "House-flies and Disease," illustrated with lantern slides. Mr. R. W. Lindsey, F.C.S., was in the chair, and Messrs. F. N. Clark, J. F. Dunstan, A. Howell, W. E. Kinsman, W. E. Miller, R. Welford, members of the Council, and J. H. Fraser and G. W. Gibson, secretaries; were present. Mr. J. Lanford Moore, chief pharmacist, St. Bart.'s, and Mrs. Moore, Mr. and Mrs. Swinton, Mrs. Frowde, the Misses Higgins, Miss Buchanan, president, Women Pharmacists' Association; Messrs. A. J. Gibbons, Smith, Lindsay, Goodall, the Misses Maughan, Hodgkinson, Turtle, and many others attended.

After Mr. Lindsey had introduced the lecturer, Dr. Higgins referred to the work done on the subject, and mentioned that much more attention was now being given to the relationship between disease and flies than in earlier centuries. The filthy habits of flies feeding on decaying matter and infecting foods with it was vividly portrayed. The fact that malaria is steadily declining in its ravages, also yellow fever, both due to mosquito and other insects tends to the hope that eventually the fly as a disease carrier will be exterminated. The process of catching flies for examination was described by Dr. Higgins, beer and sugar being used, and wool saturated with chloroform. The lecturer pointed out that there were several species of flies, domestic, blow-fly, stable-fly, etc., but of about 43,000 examined in Birmingham 70 per cent. were the housefly *Musca domestica.* Dr. Higgins suggested as remedies for the extermination of these pests effective sanitary arrangements, dust-destructors for refuse, water-closets, etc., and said that if the breeding material, garbage, etc., was burnt up the greatest incubator of flies would be destroyed. Precautions in covering all foods were strongly advised. Mention should be made of the exquisite photo-slides which showed the structure and life history of the fly, these being the work of Mr. Brailsford, of Birmingham. The lecture shows that the Council of this Association is maintaining its efficiency as an educative body.

